# Absence of XMRV in Peripheral Blood Mononuclear Cells of ARV-Treatment Naïve HIV-1 Infected and HIV-1/HCV Coinfected Individuals and Blood Donors

**DOI:** 10.1371/journal.pone.0031398

**Published:** 2012-02-13

**Authors:** Cosmina Gingaras, Bryan P. Danielson, Karen J. Vigil, Elana Vey, Roberto C. Arduino, Jason T. Kimata

**Affiliations:** 1 Section of Retrovirology, Department of Pediatrics, Baylor College of Medicine, Houston, Texas; 2 Department of Molecular Virology and Microbiology, Baylor College of Medicine, Houston, Texas; 3 Division of Infectious Diseases, Department of Internal Medicine, University of Texas Health Science Center, Houston, Texas; 4 Baylor International Pediatric AIDS Initiative, Baylor College of Medicine, Houston, Texas; National Cancer Institute, United States of America

## Abstract

**Background:**

Xenotropic murine leukemia virus-related virus (XMRV) has been found in the prostatic tissue of prostate cancer patients and in the blood of chronic fatigue syndrome patients. However, numerous studies have found little to no trace of XMRV in different human cohorts. Based on evidence suggesting common transmission routes between XMRV and HIV-1, HIV-1 infected individuals may represent a high-risk group for XMRV infection and spread.

**Methodology/Principal Findings:**

DNA was isolated from the peripheral blood mononuclear cells (PBMCs) of 179 HIV-1 infected treatment naïve patients, 86 of which were coinfected with HCV, and 54 healthy blood donors. DNA was screened for XMRV provirus with two sensitive, published PCR assays targeting XMRV *gag* and *env* and one sensitive, published nested PCR assay targeting *env*. Detection of XMRV was confirmed by DNA sequencing. One of the 179 HIV-1 infected patients tested positive for *gag* by non-nested PCR whereas the two other assays did not detect XMRV in any specimen. All healthy blood donors were negative for XMRV proviral sequences. Sera from 23 HIV-1 infected patients (15 HCV^+^) and 12 healthy donors were screened for the presence of XMRV-reactive antibodies by Western blot. Thirteen sera (57%) from HIV-1^+^ patients and 6 sera (50%) from healthy donors showed reactivity to XMRV-infected cell lysate.

**Conclusions/Significance:**

The virtual absence of XMRV in PBMCs suggests that XMRV is not associated with HIV-1 infected or HIV-1/HCV coinfected patients, or blood donors. Although we noted isolated incidents of serum reactivity to XMRV, we are unable to verify the antibodies as XMRV specific.

## Introduction

Xenotropic murine leukemia virus-related virus (XMRV) is a gammaretrovirus first discovered in the cancer-associated stroma of prostate cancer patients in 2006 [1]. More recently, XMRV DNA and infectious virions were detected in the peripheral blood mononuclear cells (PBMCs) of patients with chronic fatigue syndrome (CFS) [2]. Following these initial reports, numerous studies have either detected a very low prevalence among subjects or no XMRV at all, even in relatively large cohorts [3]–[16]. According to several studies, detection of XMRV in human specimens may be due in part to contamination of laboratory reagents or tissues with infected cell lines or murine DNA [17]–[24]. Therefore, inconsistent detection of XMRV between laboratories may be attributable to differences in the reagents used for screening for XMRV and to differences in specimen handling procedures. Additionally, it is possible that inconsistent detection of XMRV may be partly due to the unknown distribution of the virus in the human population.

XMRV may be more prevalent in the human immunodeficiency virus type 1 (HIV-1) infected population as the virus may be transmitted through the same routes as HIV-1. The ability of XMRV to infect PBMCs and its relatedness to lymphotropic mouse retroviruses suggest parenteral routes of infection, including blood transfusion and intravenous drug use [1], [2], [25], [26]. Sexual transmission has been suggested by the finding that a factor present in semen increases XMRV infectivity, and by the presence of XMRV RNA in prostatic secretions [27]. Furthermore, intravenous inoculation of Indian rhesus macaques with XMRV demonstrated persistent infection of the reproductive organs, including the prostate, cervix, vagina, and testes [26]. These findings indicate that individuals at risk for exposure to HIV-1 may also be at risk for exposure to XMRV.

The HIV-1 infected host may provide an immunological environment propitious for XMRV replication and spread. Apart from the overall deterioration of the immune system resulting largely from the depletion of CD4+ T cells (reviewed in [28]), HIV-1 encodes accessory proteins that antagonize innate antiviral host proteins shown to restrict XMRV replication, such as several members of the APOBEC3s and tetherin/BST-2 [29]–[35]. Thus, HIV-1 infected persons may potentially accommodate for XMRV replication due to suppressed immunological defenses on both systemic and cellular levels.

Based on evidence suggesting common transmission routes between HIV-1 and XMRV, and the ability of HIV-1 to neutralize immune components shown to restrict XMRV replication, we hypothesized that the prevalence of XMRV among HIV-1 infected patients may be elevated compared to healthy blood donors. In this study, we used three sensitive polymerase chain reaction (PCR) assays to screen for the presence of XMRV DNA in the PBMCs of HIV-1 infected patients, HIV-1/HCV coinfected patients, and blood donors. To increase our potential for detecting XMRV DNA in patient specimens, we used PCR assays that had been previously characterized and shown to be capable of detecting low levels of viral DNA [2], [36]. We also screened sera from a fraction of the HIV-1 and HCV infected patients, and uninfected individuals for the presence of XMRV-reactive antibodies.

## Methods

### Ethics statement

The Committee for the Protection of Human Subjects at the University of Texas Health Science Center approved the use of the PBMCs and sera for the purposes of the present study. All patient volunteers provided written informed consent. The Institutional Review Board of Baylor College of Medicine provided concurrent approval of the studies.

### Patient specimens

A total of 179 HIV-1 infected patients representing a wide range of CD4^+^ T cell counts and HIV-1 viral loads were selected for XMRV screening. Eighty six of these patients were also infected with HCV. Tests used to diagnose patients with HIV-1 infection included the COBAS AmpliPrep/COBAS TaqMan HIV-1 Test (Roche Diagnostics, Indianapolis, IN), the TRUEGENE HIV-1 Genotyping Kit (Siemens Healthcare Diagnostics, Inc., Tarrytown, NY), the GS HIV-1 Western Blot Kit (Bio-Rad Laboratories, Redmond, WA), and the Advia Centaur EHIV (Siemens Healthcare Diagnostics, Tarrytown, NY). Tests used to diagnose patients with HCV infection included the COBAS AmpliPrep/COBAS TaqMan HCV Test (Roche Diagnostics, Indianapolis, IN), the VERSANT HCV Genotype (LiPA) 2.0 Assay (Siemens Healthcare Diagnostics, Inc., Deerfield, IL), and the Advia Centaur HCV Immunoassay (Siemens Healthcare Diagnostics, Tarrytown, NY). All subjects were patients of the Thomas Street Health Center, which is an urban clinic for HIV-1 infected indigent persons run by the Harris County Hospital District in Houston, Texas. Blood was collected in EDTA vacutainers and PBMCs were isolated and stored in liquid nitrogen within 8 hours of collection according to the HIV/AIDS Network Coordination (HANC) PBMC Processing Standard Operating Procedure. In preparation for DNA isolation, the PBMC specimens were stored at −80°C (less than one month). All HIV-1 infected patients were antiretroviral treatment naïve at the time of blood collection. A total of 54 healthy blood donors from the Gulf Coast Regional Blood Center were randomly selected for XMRV screening. As with the HIV-1 infected patient specimens, PBMCs were isolated within 8 hours of blood collection and were stored at −80°C until the time of DNA extraction (less than 1 month).

### Cell culture

The LNCaP, clone FGC human prostate carcinoma cell line (ATCC no. CRL-1740), was used to produce XMRV stock and for antigen in Western blot to screen for XMRV-reactive antibodies in patient sera. Either the LNCaP cell line or the PNT1A immortalized human prostate epithelial cell line (see [37], [38]) was used to generate sensitivity controls for PCR. The LNCaP and PNT1A cell lines were cultured in RPMI medium 1640 (Invitrogen) supplemented with 10% heat-inactivated fetal bovine serum (FBS), 2 mM glutamine, 100 U/ml penicillin, and 100 µg/ml streptomycin (Invitrogen), and were propagated at 37°C with 5% CO_2_.

The R187 hybridoma cell line (ATCC no. CRL-1912, [39]) was used to generate the rat anti-spleen focus-forming virus (SFFV) p30 monoclonal antibody (mAb) that cross-reacts with XMRV p30. R187 cells were cultured in RPMI medium 1640 (Invitrogen) supplemented with 4.5 g/L D-glucose, 2.383 g/L HEPES buffer, L-glutamine, 1.5 g/L sodium bicarbonate, 110 mg/L sodium pyruvate, 0.05 mM β-mercaptoethanol, 100 U/ml penicillin, 100 µg/ml streptomycin, and 10% heat-inactivated FBS. Cell concentrations were maintained between 1×10^5^ and 1×10^6^ per ml, and the conditioned media was harvested every 3 days. Conditioned media was passed through a 0.22 µm syringe filter (BD Biosciences) and stored at −80°C.

Patient PBMCs preserved in liquid nitrogen to be activated prior to PCR screening were thawed at 37°C, washed with 9 ml of RPMI medium 1640 supplemented with 10% heat-inactivated FBS, 2 mM glutamine, 100 U/ml penicillin, and 100 µg/ml streptomycin, and re-suspended in 10 ml of the same medium with 1 µg/ml PHA (Sigma-Aldrich, St. Louis, MO). PBMCs were cultured with PHA at 37°C in 5% CO_2_ for 3 days, and then moved to the same base medium with 20 U/ml of IL-2 in place of PHA. After culturing for 4 days with IL-2, the PBMCs were collected for DNA extraction.

### DNA isolation

Genomic DNA was prepared from PBMCs using the QIAamp DNA Mini Kit (Qiagen) according to the manufacturer's instructions. DNA extractions were performed in a human tissue processing laboratory devoid of cloned XMRV or in vitro-XMRV-infected cell lines, using materials and reagents that had minimal contact with other laboratories. DNA specimens were stored at −20°C directly following isolation, in a room free of amplified or cloned DNA.

To verify the integrity of isolated DNA, the CCR5 gene was amplified using a modified version of a previously-described PCR protocol [40]. The 25 µl PCR mixtures contained: 2.5 µl GeneAmp 10× PCR Gold Buffer (Applied Biosystems), 1 mM MgCl_2_, 27 pmoles of primer CCR5c (5′-CAA AAA GAA GGT CTT CAT TAC ACC-3′), 27 pmoles of primer CCR5d (5′-CCT GTG CCT CTT CTT CTC ATT TCG-3′), 0.4 mM dNTPs, and one unit AmpliTaq Gold polymerase (Applied Biosystems). Thermocyling conditions were identical to the original protocol.

### PCR detection of XMRV

Two previously-described, non-nested oligonucleotide primer sets targeting both XMRV *gag* and *env* and one previously-described, nested oligonucleotide primer set targeting XMRV *env* were used to screen specimens for XMRV DNA (Table 1) [2], [36]. The nested *env* PCR assay was performed according to the protocol described previously, using 650 ng of template DNA [36]. All specimens were tested in triplicate with the nested *env* PCR assay. The non-nested *env* and *gag* PCR assays were modified from the original protocols. A final PCR reaction volume of 50 µl contained: 250 ng DNA, 5 µl GeneAmp 10× PCR Gold Buffer (Applied Biosystems), 2.5 mM MgCl_2_, 800 µM dNTPs, 0.3 µM of each forward and reverse primer, and 1.5 units of AmpliTaq Gold DNA polymerase (Applied Biosystems). Thermocycling conditions were as described in the original protocols [2]. All specimens were tested in triplicate with both non-nested PCR assays. All PCR reagents were mixed in a separate room, free of amplified or cloned DNA, under a containment hood that was subjected to ultra violet (UV) light before and after each round of PCRs. Sensitivity controls comprising DNA isolated from cells infected in vitro with XMRV diluted in DNA from uninfected cells were generated as described previously, with the exception that PNT1A cells (being more easily cultured) were used in place of LNCaP cells for a portion of the controls [36]. Each set of master mixes for each PCR assay used in XMRV screening was tested for sensitivity and nucleic acid contamination. Master mixes were considered adequately sensitive only if they were able to detect XMRV provirus from one infected cell diluted in 1×10^4^ uninfected cells in three of three samples using either 250 ng (non-nested PCR assays) or 650 ng (nested *env* assay) of DNA template. Master mixes were considered to be free of XMRV DNA contamination if negative results were obtained using water in place of DNA template in three of three samples. After thermocycling, 20 µl of each PCR mixture was electrophoresed through 1% agarose with ethidium bromide and visualized under UV light. PCR amplicons near to the expected size, as gauged by positive controls and molecular weight markers, were purified from agarose using the QIAEX II Gel Extraction Kit (Qiagen), according to the manufacturer's protocol. Purified amplicons were ligated into pCR2.1-TOPO using the TOPO TA Cloning Kit (Invitrogen). Plasmid constructs were used to transform either One Shot TOP10 (Invitrogen), or NEB 10-beta (New England BioLabs) chemically competent *Escherichia coli*, isolated with the QIAPrep Spin Miniprep Kit (Qiagen), and the sequences of the inserted DNA fragments were determined. A patient-derived XMRV *gag* sequence was deposited into GenBank under accession number JN235142.

**Table 1:** Primers used for screening PBMC DNA specimens for XMRV.

Target	Ref.	Primer	Sequence	Location^a^
XMRV *gag*	[2]	Forward	5′-ATCAGTTAACCTACCCGAGTCGGAC-3′	424–448
		Reverse	5′-GCCGCCTCTTCTTCATTGTTCTC-3′	1132–1154
XMRV *env*	[2]	Forward	5′- GCTAATGCTACCTCCCTCCTGG-3′	5922–5943
		Reverse	5′-GGAGCCCACTGAGGAATCAAAACAGG-3′	6247–6272
XMRV *env*	[36]	Forward	5′-ACCAGACTAAGAACTTAGAACCTCG-3′	5609–5633
		Reverse	5′-AGCTGTTCAGTGATCACGGGATTAG-3′	6472–6496
		Forward	5′-GAACAGCATGGAAAGTCCAGCGTTC-3′	5747–5771
		Reverse	5′-CAGTGGATCGATACAGTCTTAGTCC-3′	6375–6399

a Location of 5′ end of forward primer target site to 3′ end of reverse primer target site on XMRV VP62 reference genome (accession no. DQ399707.1).

### PCR screening for contaminants

A previously-described PCR assay targeting intracisternal A-type particle long terminal repeats (IAPs) was used to screen specimens for murine DNA contamination [17]. All PCR reagents and conditions were identical to those described previously, with the exception that 250 ng of DNA template was used in place of 200 ng for screening the PBMC DNA specimens [17]. DNA isolated from a vial of preserved murine EL4 cells (ATCC no. TIB-39), kindly provided by Dr. Qizhi C. Yao, was used as a positive control. DNA from EL4 cells was isolated and stored in a laboratory separate from both the laboratory where subject specimens were stored and the laboratory where PCRs were conducted, in order to minimize chances for contamination. Sensitivity was determined by screening dilutions of murine EL4 cell DNA in a background of both 200 and 250 ng of LNCaP DNA. Six picograms was considered to be the mass of DNA in one murine cell (one cell equivalent). For each master mix used for screening subject specimens for murine DNA contamination, five positive controls and three negative controls were included. The positive controls consisted of PCRs using templates of 60, 6, 0.6, and 0.06 pg of EL4 DNA in a background of 250 ng LNCaP DNA, as well as 6 pg of EL4 DNA without background DNA. The negative controls consisted of PCRs with either water or LNCaP (both XMRV-infected and uninfected) DNA in place of template DNA. Following thermocycling, the PCR mixtures were electrophoresed through 1.5% agarose with ethidium bromide and were visualized under UV light.

A previously-described nested PCR assay was used to screen for the VP62 XMRV plasmid as a contaminant in patient specimens [36]. The PCR assay amplifies a DNA fragment spanning the junction between the pCDNA3.1(−) (Invitrogen) multiple cloning site and the 5′ terminus of XMRV strain VP62. This assay was previously found to be capable of detecting 10 VP62 plasmids diluted in 600 ng of LNCaP DNA in three of three samples and one plasmid in the same amount of LNCaP DNA in one of three samples [36]. Thermocycling conditions for the first-round PCR were as follows: 95°C for 5 min; 35 cycles of 94°C for 30 sec, 56°C for 1.5 min, 72°C for 1.5 min; and ending with 72°C for 10 min. Thermocycling conditions for the second-round reaction were the same as the first round, with the exception that the annealing temperature (56°C) was changed to 52°C. DNA specimens were screened in one reaction using 250 ng of template DNA and in an additional reaction using 600 ng of template DNA.

### Western blot

Rat monoclonal Ab to SFFV p30 generated from the R187 hybridoma cell line was used as a positive control for detection of XMRV p30 capsid in Western blot [39]. Affinity-purified rabbit polyclonal antibodies (pAb) to the peptide sequence, DDPEPDIGDGCRSPGGRKR, corresponding to a region of XMRV gp70 were generated by Open Biosystems, Inc. (Thermo Fisher Scientific). These antibodies were used as a positive control for detection of XMRV gp70 in Western blot. To screen serum specimens for XMRV reactive antibodies, XMRV-infected LNCaP cells were lysed in buffer containing 50 mM tris-HCl, 150 mM NaCl, 1% NP-40, 0.5% sodium deoxycholate, 1% sodium dodecyl sulfate (SDS), and the Complete Mini EDTA-free protease inhibitor cocktail (Roche). Cell lysates were electrophoresed through 15% Tris-HCl Criterion precast gels (Bio-Rad Laboratories) and transferred to Amersham Hybond-ECL nitrocellulose membranes (GE Healthcare). Membrane sections were blocked with 5% nonfat dry milk, and incubated with 1∶1,000 diluted R187 cell supernatant (anti-CA), anti-XMRV gp70 pAb (anti-Env), or 1∶100 diluted patient serum. After washing, membranes were incubated with secondary antibodies conjugated to horseradish peroxidase: rabbit anti-rat IgG (Sigma-Aldrich), goat anti-rabbit IgG (Santa Cruz Biotechnology), or goat anti-human IgG (γ-chain specific, Sigma-Aldrich). Membranes were washed again, incubated briefly with Pierce ECL Western Blotting Substrate (Thermo Scientific) and exposed to HyBlot CL autoradiography film (Denville Scientific).

## Results

### PCR validation

**Figure 1:**
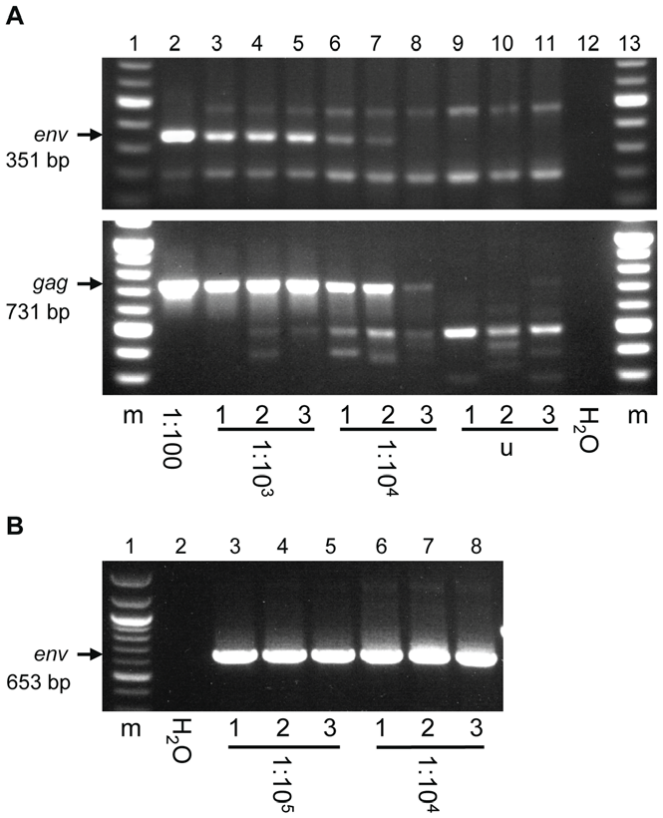
Sensitivity analysis of XMRV PCR assays. PCR products were analyzed on agarose gels containing ethidium bromide. (A) Non-nested PCR assays targeting the XMRV *env* gene (top panel) and the *gag* gene (bottom panel), and (B) a nested PCR assay targeting the XMRV *env* gene were evaluated for their ability to detect either (A) provirus in XMRV-infected PNT1A cell DNA or (B) provirus in XMRV-infected LNCaP cell DNA diluted in uninfected cell DNA. Dilutions of infected cells in uninfected cells are indicated by ratios, i.e. 1∶10^4^ indicates one infected cell diluted in 10^4^ uninfected cells. (m) 100 base pair molecular weight marker, (H_2_O) water used in place of DNA template as a negative control, (u) uninfected PNT1A DNA used as template for negative control.

Detection of XMRV (and related viruses) with PCR-based methods has proven to be rather difficult, with reports of low frequencies of provirus-containing cells [15], [36], [41] and the inability to amplify multiple regions of the XMRV genome from the same specimen [8], [25], [36]. In order to maximize the probability of detecting XMRV by PCR in patient specimens that harbor provirus, we decided to use three different published assays that have been successful in earlier studies (Table 1). Two non-nested primer sets developed by Lombardi et al. were shown to be capable of detecting XMRV *gag* and *env* in the PBMCs of chronic fatigue syndrome patients, whereas a nested PCR assay developed in our laboratory had been used to detect XMRV *env* in the prostatic tissue of prostate cancer patients [2], [36]. Prior to screening patient samples, we tested the sensitivity of the three primers sets. We found the non-nested *env* primers capable of detecting one infected cell diluted in 1×10^4^ uninfected cells in two of three samples using 250 ng of template DNA (∼4.2×10^4^ cells, Figure 1A, top panel). The non-nested *gag* primers were found to be capable of detecting the same dilution of infected cells in three of three samples (Figure 1A, bottom panel). Considerable non-specific amplification was seen for both assays, especially for *gag*, which occasionally included background amplification products near to the size expected for the target sequence (Figure 1A, bottom panel, lane 11). The nested *env* primers were found to be capable of detecting one infected cell per 1×10^5^ uninfected cells in three of three samples using 650 ng of template DNA (∼1×10^6^ cells, Figure 1B). We contend that, when used in combination, these three PCR assays are likely to detect low levels of XMRV sequence because they target multiple genes, have a high degree of sensitivity, and are reported to have been successful.

### XMRV DNA in patient PBMCs

**Figure 2:**
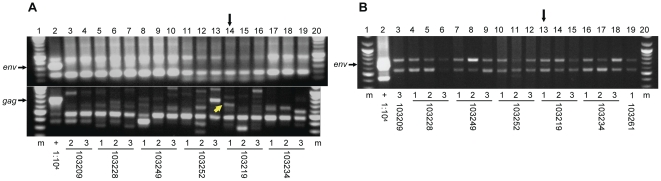
Screening for XMRV in patient PBMCs by PCR. PCR products were analyzed on agarose gels containing ethidium bromide. (A) Representative gels for non-nested *env* (top panel) and non-nested *gag* (bottom panel) PCRs are shown containing a set of three replicates for each of 5 HIV-1^+^ patient samples. A yellow arrow indicates the sole PCR band, from patient 103219, found to be comprised of XMRV DNA by sequencing. (B) A representative gel for nested *env* PCR is shown for the same 5 HIV-1^+^ patient samples depicted in (A). Vertical black arrows in (A) and (B) indicate lanes from patient 103219 containing either (A, bottom panel) a band comprised of XMRV sequence or (B) a band of the expected mobility for the target sequence. (m) 100 base pair molecular weight marker, (1∶10^4^) DNA from one infected cell diluted in DNA from 10^4^ uninfected cells used as template for positive control.

To determine whether the prevalence of XMRV is elevated among HIV-1 infected and HIV-1/HCV coinfected individuals compared to healthy blood donors, we screened for XMRV *gag* and *env* genes in DNA isolated from the PBMCs of 179 HIV-1^+^ individuals, including 86 coinfected with HCV, and in DNA from 54 healthy blood donors. Each DNA specimen was screened by PCR in triplicate with each of the three primer sets listed in Table 1. Consistent with the original protocols for each assay, 250 ng of DNA template was used for the non-nested PCRs, whereas 650 ng of template was used for the nested *env* PCR. We found that both primers sets targeting the *env* gene produced a few non-specific amplification products, but rarely any that were of the expected size for the target sequence (Figure 2A, top panel; Figure 2B). The few products that were of the expected size were cloned and sequenced but found to be human chromosomal sequence artifacts, i.e. bands in Figure 2A, top panel, lanes 5–7. The non-nested *gag* primers were found to produce many non-specific amplification products that were frequently near to the expected size for the target sequence (Figure 2A, bottom panel). We cloned and sequenced most non-nested *gag* PCR products close to the expected size. We were unable to clone a few PCR amplicons, which produced exceedingly-faint bands when observed on an agarose gel. All successfully cloned products were not XMRV proviral DNA sequences with one exception (Figure 2A, bottom panel, lane 14). The expected 731 nucleotide *gag* product (GenBank accession no. JN235142) from one of three PCR replicates for HIV-1/HCV coinfected patient 103219 was found to be identical to the sequence of the XMRV plasmid clone, VP62 (GenBank accession no. DQ399707.1). In order to test whether our detection of XMRV in patient 103219 was an artifact of plasmid contamination, we screened this DNA specimen with a highly sensitive, nested PCR assay specific to VP62 [36]. No amplification products were observed in triplicate PCRs using both 250 and 650 ng of template DNA (data not shown). Patient 103219 tested negative for XMRV by non-nested *env* PCR, and although we did observe a light band of the expected size in one of three PCR replicates of nested *env* PCR, attempts to clone and sequence this product were unsuccessful (Figure 2B, lane 13). All 54 blood donors tested negative for XMRV with all three PCR assays. For these specimens, cloning and sequencing revealed that all PCR products near to the expected size on an agarose gel were not XMRV. Negative controls with water in place of DNA were included with every batch of PCRs and never produced any amplified DNA products throughout the study. The PCR screening results for XMRV provirus in the PBMCs of the patients and donors are summarized in Table 2.

**Table 2:** Summary of XMRV screening results.

		*Gag* PCR	*Env* PCR		Anti-XMRV Ab^b^	
Subjects	Status	Non-nested	Non-nested	Nested	Gag	Env
Patients	HIV-1^+^, HCV^−^	0/93^a^	0/93	0/93	5/8	0/8
	HIV-1^+^, HCV^+^	1/86	0/86	0/86	7/15	1/15
Donors	HIV-1^−^, HCV^−^	0/54	0/54	0/54	6/12	0/12

a Fractions are: number of subjects scoring positive/total number of subjects screened.

b Ab, antibody.

Stimulation and culturing of patient PBMCs was reported to have increased the sensitivity of the non-nested *gag* and *env* PCR assays for detection of XMRV [42]. Therefore, we stimulated PBMC specimens from 5 HIV-1 infected patients and from 5 HIV-1/HCV coinfected patients with PHA and IL-2 and cultured them for a week prior to DNA isolation. We screened for XMRV in both stimulated and unstimulated PBMCs from each of the 10 patients with all three PCR assays using the same protocol as with the other 169 patient specimens. All 10 patients tested negative (data not shown).

**Figure 3:**
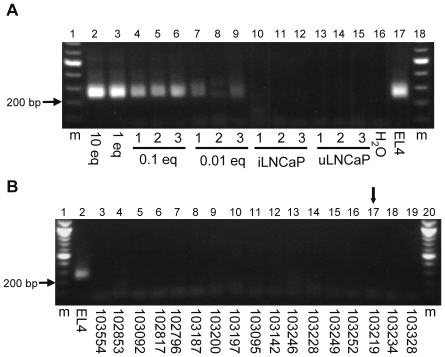
Detecting murine DNA by IAP PCR. PCR products were analyzed on 1.5% agarose gels containing ethidium bromide. (A) Sensitivity of the IAP PCR assay was determined by performing PCRs on titrations of EL4 murine cell line DNA in a background of 200 ng LNCaP DNA. One murine cell equivalent (1 eq) indicates 6 pg of EL4 DNA. XMRV-infected LNCaP (iLNCaP) and uninfected LNCaP (uLNCaP) were included as controls. (B) Screening results for 17 HIV-1^+^ patient samples. Arrow points to sample 103219, which tested positive for XMRV by non-nested gag PCR. (m) 100 base pair molecular weight marker, (EL4) 6 pg of murine EL4 cell line DNA without a background of human DNA.

Apart from the relatedness of XMRV to murine retroviruses, a murine retroviral origin for XMRV sequences has been suggested in recent work by Paprotka et al. [20]. Furthermore, several recent reports have shown that minute quantities of murine DNA in subject specimens or laboratory reagents can lead to false-positives when using PCR-based methods to screen for XMRV [17], [21]–[24]. Therefore, we screened for the presence of murine DNA contamination using the PCR method described by Oakes et al. [17]. This PCR assay targets murine retrotransposons (IAPs), which are estimated to be present at a copy number of approximately 1×10^3^ per mouse cell [43], [44]. Prior to screening subject specimens, we tested the sensitivity of the IAP PCR assay. In our hands, the IAP PCR assay was found capable of detecting 1/100^th^ of the DNA present in a single mouse cell diluted in a background of 200 ng of LNCaP cell DNA in three of three samples (Figure 3A). Using this sensitive assay, we screened a subset of 38 PBMC DNA specimens from the HIV-1 infected and HIV-1/HCV coinfected patients, which were selected on the basis that they were either positive for XMRV by sequencing, or they produced a PCR band close to the expected size on an agarose gel by any of the three PCR assays used to screen for XMRV. All 38 specimens tested negative for the presence of IAPs, ruling out murine DNA contaminants as a source for the XMRV sequence detected in patient 103219 (Figure 3B). Thus, in 1 of three PCR assays, 1 of 179 HIV-1^+^ patients (1 of 86 HIV-1/HCV coinfected patients) tested positive for XMRV (in one of three PCR replicates targeting *gag*) and all 54 healthy blood donors tested negative. The minimal detection of XMRV in this cohort is in line with multiple other studies that have screened for XMRV in HIV-1 infected cohorts [16], [45]–[50]. Due to the exact match of the XMRV sequence derived from patient 103219 with the VP62 XMRV plasmid clone used in our laboratory, and to the inability of the two other PCR assays to produce clonable amplicons of the expected size, we cannot conclude that DNA from patient 103219 harbored either XMRV provirus or a trace amount of VP62 plasmid contamination that was missed by the VP62 plasmid-specific nested PCR assay. The results of the PCR screen for XMRV do not support an association between XMRV and HIV-1 or HCV infections.

### XMRV-reactive antibodies in sera

**Figure 4:**
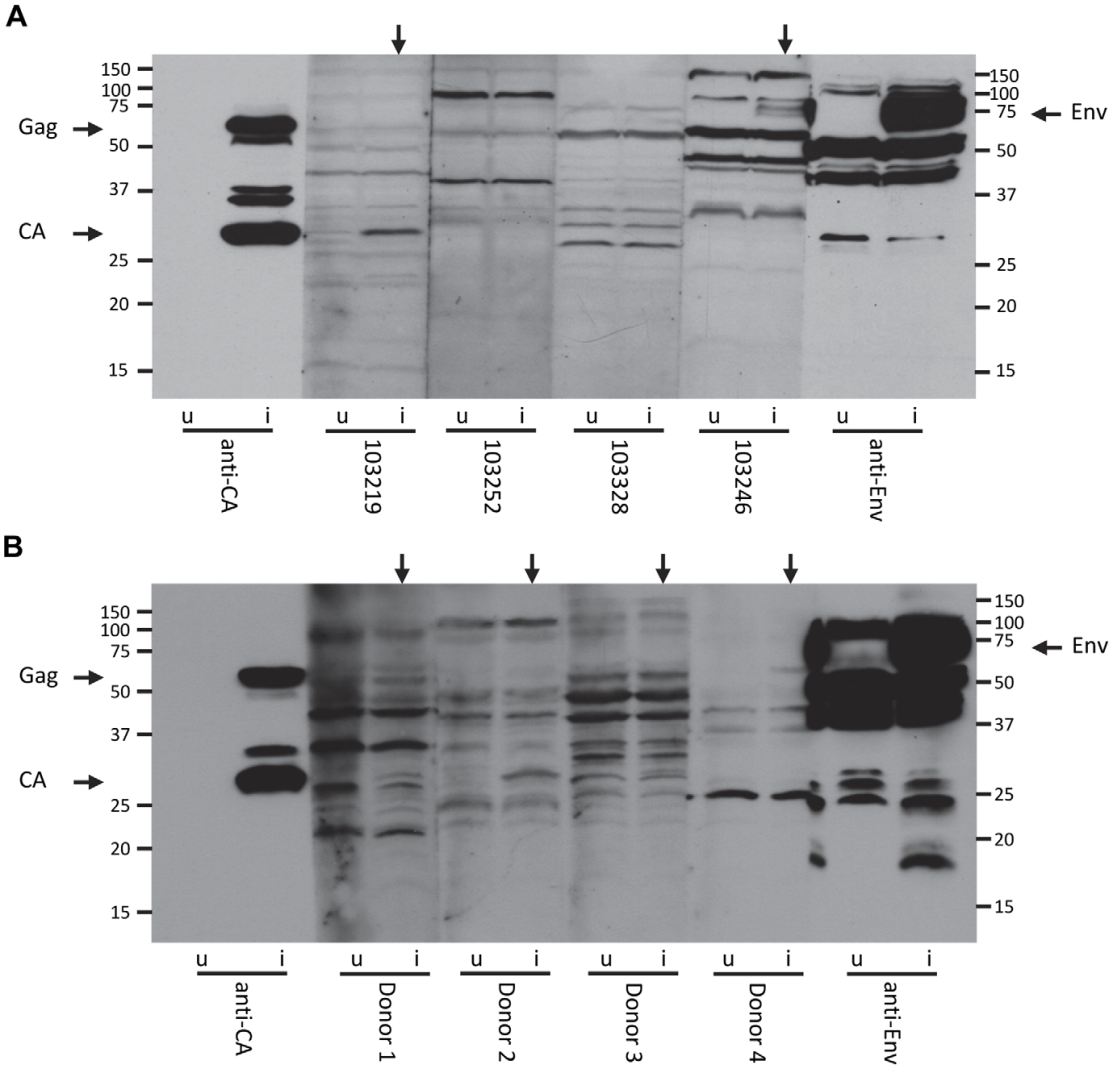
XMRV-reactive antibodies in patient and healthy blood donor sera. Representative Western blots using uninfected (u) and XMRV-infected (i) LNCaP cell lysate as antigen for (A) HIV-1 infected patient sera or (B) healthy blood donor sera, and positive-control antibodies against p30 capsid (anti-CA) and gp70 SU (anti-Env). (A) Vertical arrows indicate lanes in which patient sera displayed reactivity to either XMRV capsid (left arrow, 103219) or XMRV envelope (right arrow, 103246). (B) Vertical arrows indicate lanes in which blood donor sera displayed reactivity to XMRV capsid (all four donors on the blot shown). Protein mobilities are indicated in kiloDaltons.

To further search for evidence of XMRV in the HIV-1 infected and HIV-1/HCV coinfected patients, we screened for the presence of XMRV-reactive antibodies in 23 of the 179 HIV-1^+^ and HIV-1^+^/HCV^+^ subjects and in 12 additional healthy blood donors. The 23 HIV-1^+^ (15 HCV^+^) patients to be tested for XMRV-reactive antibodies were chosen if either non-nested *gag* or *env* PCR amplified a product near to the expected mobility on agarose. Equivalent amounts of whole-cell lysate from uninfected and XMRV VP62-infected LNCaP prostate carcinoma cells were used as antigen for testing sera from each patient by immunoblotting. Signals due to background reactivity signify similar levels of proteins present for both uninfected and XMRV-infected LNCaP cell lysates (Figure 4). Interestingly, we obtained signals from 13 of the 23 patient sera on XMRV-infected cell lysate corresponding to the mobility of either the capsid or Env proteins that were not present for uninfected cell lysate (Figure 4A and Figure S1). Five of eight sera from HIV-1^+^ patients were reactive to XMRV whereas 8 of 15 sera from HIV-1^+^/HCV^+^ patients were reactive (Table 2). Of the 13 XMRV-reactive sera, 12 contained capsid-reactive antibodies, and one contained Env-reactive antibodies (Table 2). Reactivity to XMRV capsid was observed for patient 103219 (Figure 4A). Similar to the HIV-1^+^ and HIV-1^+^/HCV^+^ patient sera, reactivity to XMRV was seen for 6 of the 12 healthy blood donors (Figure 4B and Figure S2). For the healthy blood donors, reactivity was only observed for XMRV capsid (Table 2). The greater ability to detect capsid-reactive antibodies compared to Env-reactive antibodies has been reported previously for plasma from healthy donors and prostate cancer and chronic fatigue syndrome (CFS) patients [5]. Although these data are suggestive of infection, without serum from a confirmed XMRV-infected individual, it is unclear whether reactivity from these 13 patient sera represents a true adaptive immune response against XMRV or is simply due to the presence of cross-reactive antibodies.

## Discussion

We screened DNA isolated from the PBMCs of 179 HIV-1 infected patients, including 86 coinfected with HCV, and 54 blood donors for the presence of XMRV provirus. Only one study participant (HIV-1^+^/HCV^+^) tested positive for XMRV *gag* sequence in our PCR screen despite the use of three sensitive, published assays that have reportedly been successful at detecting XMRV in different human cohorts [2], [36]. The low frequency of XMRV detected in this study is in line with other reports in which no XMRV was detected in separate HIV-1 infected cohorts [16], [45]–[50]. Notably, all but one [49] study searching for XMRV in HIV-1 infected cohorts to date have screened for the virus in the blood or in constituents of the blood. While the agreement in results among reports regarding the prevalence of XMRV in HIV-1 infected cohorts may indicate that XMRV is largely absent from this population as a whole, it is also possible that XMRV resides primarily in a cellular compartment other than blood. On this note, it is important to point out that all but a few [2], [5], [25] reports on screens for XMRV in the blood or in blood constituents were unable to detect the virus [3], [4], [12], [13], [16], [45]–[48], [50]–[52]. Comparatively, more studies detect XMRV, at least at a low prevalence [1], [8], [11], [14], [15], [36], [41], [53], [54], than those that do not [6], [9], [10], [49], [51], when *non-blood* tissue specimens are screened. As most reports on screens for XMRV in non-blood-derived specimens pertain to prostate cancer cohorts, it is unclear whether disease status or the type of tissue screened is the main determinant for detection of the virus. A clue may be provided in a recent report on the kinetics and dissemination of XMRV in Indian rhesus macaques after intravenous inoculation [26]. In that study, XMRV provirus became undetectable in macaque PBMCs after only one month post-inoculation, whereas provirus could be detected from other macaque tissues throughout the 291 day duration of the study [26]. If XMRV provirus is cleared from the blood one month after infection of humans, then the blood (or its constituents) is not a reliable tissue compartment to screen when attempting to establish the prevalence of the virus.

In light of the difficulty of detecting XMRV, which may be partially attributable to lack of knowledge regarding tissue tropism in the human host, it is important to use multiple methods for screening. This is also important due to the pitfalls and limitations associated with certain methods. A drawback to employing the use of sensitive PCR-based techniques over others in screening studies is the relative ease at which contamination may lead to false-positives. The single XMRV *gag* sequence detected in the PBMC DNA of an HIV-1/HCV coinfected study participant (103219) was identical to the *gag* gene of the VP62 XMRV plasmid clone used in our laboratory, raising the possibility that the patient-derived sequence represents an artifact of plasmid contamination. Our triplicate negative PCR controls included in every batch of specimens screened never amplified a DNA product throughout the study. Furthermore, we found no evidence for plasmid contamination in patient 103219 by nested PCR screening. However, since the non-nested and nested PCR assays targeting XMRV *env*, as well as repeated rounds of the non-nested *gag* PCR, were all unable to produce clonable amplicons of the expected size, we find it difficult to conclude that DNA from patient 103219 contained XMRV provirus.

Antiretroviral drugs used in highly effective anti-HIV-1 combinations have been shown to inhibit XMRV replication in vitro [55], [56]. Since most studies on XMRV in HIV-1 infected cohorts screened patients treated with antiretroviral medications, it is possible that the virus had largely been missed in this demographic [45], [46], [48]–[50]. However, our PCR screening for XMRV was essentially negative, despite having tested a population that was entirely treatment naïve. This is in line with the results of other studies that have screened treatment naïve HIV-1^+^ subjects and suggests that XMRV may be largely undetectable in the blood of HIV-1 infected persons regardless of their treatment status [16], [45], [47]–[50].

In light of the minimal detection of XMRV DNA in the PBMCs of the subjects we tested, it is possible that the screening methodology employed in this study was not sensitive enough to detect low levels of provirus that may have been present. We find this unlikely due to our use of three different PCR assays that have been shown to be very sensitive and effective at detecting XMRV in patient specimens, and that target different locations on the viral genome (Table 1). For the non-nested PCR assays, however, it was reported that sensitivity for detecting XMRV in PBMCs could be increased if the PBMCs are stimulated with PHA and cultured in IL-2-containing media prior to PCR [42]. Despite stimulating and culturing the PBMCs of 10 HIV-1^+^ patients (5 HCV^+^) we found no evidence of XMRV infection upon PCR screening with any of the three assays.

We found that the non-nested *gag* PCR assay amplified a stretch of human genomic sequence that is almost precisely the same length as the intended proviral target sequence, leading to a high rate of false-positive PCR products when viewed on an agarose gel. When testing patient DNA with the *gag* PCR assay using the HotStart-IT FideliTaq polymerase (Affymetrix), which was used in the original protocol by Lombardi and colleagues, we still observed amplification of human genomic sequence of the length expected for the proviral target sequence (data not shown).

Our screen of sera from a subset of 23 of the HIV-1 infected patients detected antibodies reactive to proteins of the expected mobility for XMRV capsid, Gag polyprotein, or Env in 13 (56%) samples. Interestingly, only one of these 13 sera was reactive to envelope (Table 2). These results are in general agreement with a previous report in which only antibodies reactive to XMRV capsid were detected in the plasma of patients with CFS and prostate cancer [5]. Seroreactivity to XMRV-infected cell lysate was split almost evenly between HIV-1^+^ and HIV-1^+^/HCV^+^ patients with rates of 5/8 (62.5%) and 8/15 (53%) for each, respectively (Table 2). Similar to the HIV-1^+^ patients, we detected antibodies reactive to XMRV capsid in 6 of 12 (50%) sera from healthy blood donors, indicating no difference in rates of reactivity to XMRV between the two groups (Table 2). It is possible that the positive signals obtained in our immunoblots are due to the presence of cross-reactive antibodies to proteins encoded by human endogenous retroviruses (HERVs), a large group of which is similar to MLVs [57], [58]. Human IgG reactivity to MLV capsid has been reported previously [59], [60]. In one study, a higher frequency of individuals with MLV capsid-reactive IgG was seen with HIV-1 infection compared to HIV-1 negative controls, a trend we did not observe with this cohort [59]. Nonetheless, proteins encoded by HERVs represent a potential source of antigen that may give rise to antibodies that are cross-reactive with XMRV. Alternatively, the XMRV-reactive antibodies detected in the sera of the HIV-1^+^ and HIV-1^+^/HCV^+^ and healthy subjects may have been elicited by an infection with XMRV or another related exogenous virus that had been cleared from the PBMCs prior to the time of blood collection, suggesting a latent infection in a tissue compartment other than blood as previously found in experimental infection of rhesus macaques [26]. The lack of an antibody that has proven specificity for XMRV has led to inconclusive results when using antibody-based screening methods. For example, it was recently discovered that human T-cell leukemia virus (HTLV) infection can elicit antibodies that are cross-reactive with XMRV p15E due to a homologous region on HTLV gp21 [61].

In conclusion, the results of our screen of HIV-1 infected, HIV-1/HCV coinfected, and uninfected subjects do not support an association between XMRV and HIV-1 or HCV infections. Our report adds to accumulating evidence from other studies conducted around the world, not only against an association between these viral infections, but also against the presence of XMRV in the blood.

## Supporting Information

Figure S1 **Screen for XMRV-reactive antibodies in HIV-1^+^ and HIV-1^+^/HCV^+^ patient sera.** Western blots using uninfected (u) and XMRV-infected (i) LNCaP cell lysate as antigen for patient sera and positive-control antibodies against p30 capsid (anti-CA) and gp70 SU (anti-Env). Vertical arrows indicate lanes in which patient sera displayed reactivity to XMRV capsid. Protein mobilities are indicated in kiloDaltons. Vertical arrows with asterisks indicate lanes in which signals for XMRV-reactivity are more apparent with a longer film exposure.
(TIF)

Figure S2 **Screen for XMRV-reactive antibodies in healthy blood donors.** Western blots using uninfected (u) and XMRV-infected (i) LNCaP cell lysate as antigen for healthy blood donor sera and positive-control antibodies against p30 capsid (anti-CA) and gp70 SU (anti-Env). Vertical arrows indicate lanes in which patient sera displayed reactivity to XMRV capsid. Protein mobilities are indicated in kiloDaltons.
(TIF)

## References

[1] Urisman A, Molinaro RJ, Fischer N, Plummer SJ, Casey G (2006). Identification of a Novel Gammaretrovirus in Prostate Tumors of Patients Homozygous for R462Q *RNASEL* Variant.. PLoS Pathog.

[2] Lombardi VC, Ruscetti FW, Das Gupta J, Pfost MA, Hagen KS (2009). Detection of an Infectious Retrovirus, XMRV, in Blood Cells of Patients with Chronic Fatigue Syndrome.. Science.

[3] Groom H, Boucherit V, Makinson K, Randal E, Baptista S (2010). Absence of xenotropic murine leukaemia virus-related virus in UK patients with chronic fatigue syndrome.. Retrovirology.

[4] Hong P, Li J, Li Y (2010). Failure to detect Xenotropic murine leukaemia virus-related virus in Chinese patients with chronic fatigue syndrome.. Virology Journal.

[5] Furuta R, Miyazawa T, Sugiyama T, Kuratsune H, Ikeda Y (2011). No association of xenotropic murine leukemia virus-related virus with prostate cancer or chronic fatigue syndrome in Japan.. Retrovirology.

[6] Hohn O, Krause H, Barbarotto P, Niederstadt L, Beimforde N (2009). Lack of evidence for xenotropic murine leukemia virus-related virus (XMRV) in German prostate cancer patients.. Retrovirology.

[7] Switzer W, Jia H, Hohn O, Zheng H, Tang S (2010). Absence of evidence of Xenotropic Murine Leukemia Virus-related virus infection in persons with Chronic Fatigue Syndrome and healthy controls in the United States.. Retrovirology.

[8] Switzer WM, Jia H, Zheng H, Tang S, Heneine W (2011). No Association of Xenotropic Murine Leukemia Virus-Related Viruses with Prostate Cancer.. PLoS ONE.

[9] Aloia AL, Sfanos KS, Isaacs WB, Zheng Q, Maldarelli F (2010). XMRV: A New Virus in Prostate Cancer?. Cancer Res.

[10] Sakuma T, Hue S, Squillace K, Tonne J, Blackburn P (2011). No evidence of XMRV in prostate cancer cohorts in the Midwestern United States.. Retrovirology.

[11] Fischer N, Hellwinkel O, Schulz C, Chun FKH, Huland H (2008). Prevalence of human gammaretrovirus XMRV in sporadic prostate cancer.. Journal of Clinical Virology.

[12] Erlwein O, Kaye S, McClure MO, Weber J, Wills G (2010). Failure to Detect the Novel Retrovirus XMRV in Chronic Fatigue Syndrome.. PLoS ONE.

[13] Kuppeveld FJM, de Jong AS, Lanke KH, Verhaegh GW, Melchers WJG (2010). Prevalence of xenotropic murine leukaemia virus-related virus in patients with chronic fatigue syndrome in the Netherlands: retrospective analysis of samples from an established cohort.. BMJ.

[14] Martinez-Fierro M, Leach R, Gomez-Guerra L, Garza-Guajardo R, Johnson-Pais T (2010). Identification of viral infections in the prostate and evaluation of their association with cancer.. BMC Cancer.

[15] Verhaegh GW, de Jong AS, Smit FP, Jannink SA, Melchers WJG (2011). Prevalence of human xenotropic murine leukemia virus-related gammaretrovirus (XMRV) in dutch prostate cancer patients.. Prostate.

[16] Henrich TJ, Li JZ, Felsenstein D, Kotton CN, Plenge R (2010). Xenotropic Murine Leukemia Virus-Related Virus Prevalence in Patients with Chronic Fatigue Syndrome or Chronic Immunomodulatory Conditions.. Journal of Infectious Diseases.

[17] Oakes B, Tai A, Cingoz O, Henefield M, Levine S (2010). Contamination of human DNA samples with mouse DNA can lead to false detection of XMRV-like sequences.. Retrovirology.

[18] Hue S, Gray E, Gall A, Katzourakis A, Tan C (2010). Disease-associated XMRV sequences are consistent with laboratory contamination.. Retrovirology.

[19] Garson J, Kellam P, Towers G (2011). Analysis of XMRV integration sites from human prostate cancer tissues suggests PCR contamination rather than genuine human infection.. Retrovirology.

[20] Paprotka T, Delviks-Frankenberry KA, Cingoz O, Martinez A, Kung HJ (2011). Recombinant Origin of the Retrovirus XMRV.. Science.

[21] Robinson M, Erlwein O, Kaye S, Weber J, Cingoz O (2010). Mouse DNA contamination in human tissue tested for XMRV.. Retrovirology.

[22] Sato E, Furuta R, Miyazawa T (2010). An Endogenous Murine Leukemia Viral Genome Contaminant in a Commercial RT-PCR Kit is Amplified Using Standard Primers for XMRV.. Retrovirology.

[23] Tuke PW, Tettmar KI, Tamuri A, Stoye JP, Tedder RS (2011). PCR Master Mixes Harbour Murine DNA Sequences.. Caveat Emptor! PLoS ONE.

[24] Shin CH, Bateman L, Schlaberg R, Bunker AM, Leonard CJ (2011). Absence of XMRV Retrovirus and Other Murine Leukemia Virus-Related Viruses in Patients with Chronic Fatigue Syndrome.. J Virol.

[25] Lo SC, Pripuzova N, Li B, Komaroff AL, Hung GC (2010). Detection of MLV-related virus gene sequences in blood of patients with chronic fatigue syndrome and healthy blood donors.. Proceedings of the National Academy of Sciences.

[26] Onlamoon N, Das Gupta J, Sharma P, Rogers K, Suppiah S (2011). Infection, viral dissemination and antibody responses of Rhesus macaques exposed to the human gammaretrovirus XMRV.. J Virol.

[27] Hong S, Klein EA, Das Gupta J, Hanke K, Weight CJ (2009). Fibrils of Prostatic Acid Phosphatase Fragments Boost Infections with XMRV (Xenotropic Murine Leukemia Virus-Related Virus), a Human Retrovirus Associated with Prostate Cancer.. J Virol.

[28] Fauci AS (1988). The Human Immunodeficiency Virus: Infectivity and Mechanisms of Pathogenesis.. Science.

[29] Bogerd HP, Zhang F, Bieniasz D, Cullen BR (2011). Human APOBEC3 proteins can inhibit xenotropic murine leukemia virus-related virus infectivity.. Virology.

[30] Paprotka T, Venkatachari NJ, Chaipan C, Burdick R, Delviks-Frankenberry KA (2010). Inhibition of Xenotropic Murine Leukemia Virus-Related Virus by APOBEC3 Proteins and Antiviral Drugs.. J Virol.

[31] Groom HCT, Yap MW, Galao RP, Neil SJD, Bishop KN (2010). Susceptibility of xenotropic murine leukemia virus-related virus (XMRV) to retroviral restriction factors.. Proceedings of the National Academy of Sciences.

[32] Stieler K, Fischer N (2010). Apobec 3G Efficiently Reduces Infectivity of the Human Exogenous Gammaretrovirus XMRV.. PLoS ONE.

[33] Marin M, Rose KM, Kozak SL, Kabat D (2003). HIV-1 Vif protein binds the editing enzyme APOBEC3G and induces its degradation.. Nat Med.

[34] Neil SJD, Zang T, Bieniasz PD (2008). Tetherin inhibits retrovirus release and is antagonized by HIV-1 Vpu.. Nature.

[35] Sheehy AM, Gaddis NC, Choi JD, Malim MH (2002). Isolation of a human gene that inhibits HIV-1 infection and is suppressed by the viral Vif protein.. Nature.

[36] Danielson BP, Ayala GE, Kimata JT (2010). Detection of Xenotropic Murine Leukemia Virus-Related Virus in Normal and Tumor Tissue of Patients from the Southern United States with Prostate Cancer Is Dependent on Specific Polymerase Chain Reaction Conditions.. Journal of Infectious Diseases.

[37] Degeorges A, Hoffschir F, Cussenot O, Gauville C, Le Duc A (1995). Recurrent cytogenetic alterations of prostate carcinoma and amplification of *c-myc* or epidermal growth factor receptor in subclones of immortalized pnt1 human prostate epithelial cell line.. International Journal of Cancer.

[38] Cussenot O (1991). Immortalization of human adult normal prostatic epithelial cells by liposomes containing large T-SV40 gene.. The Journal of urology.

[39] Chesebro B, Britt W, Evans L, Wehrly K, Nishio J (1983). Characterization of monoclonal antibodies reactive with murine leukemia viruses: Use in analysis of strains of friend MCF and friend ecotropic murine leukemia virus.. Virology.

[40] Huang Y, Paxton WA, Wolinsky SM, Neumann AU, Zhang L (1996). The role of a mutant CCR5 allele in HIV-1 transmission and disease progression.. Nat Med.

[41] Schlaberg R, Choe DJ, Brown KR, Thaker HM, Singh IR (2009). XMRV is present in malignant prostatic epithelium and is associated with prostate cancer, especially high-grade tumors.. Proceedings of the National Academy of Sciences.

[42] Mikovits JA, Lombardi VC, Pfost MA, Hagen KS, Ruscetti FW (2010). Detection of an infectious retrovirus, XMRV, in blood cells of patients with chronic fatigue syndrome.. Virulence.

[43] Dupressoir A, Heidmann T (1997). Expression of intracisternal A-particle retrotransposons in primary tumors of oncogene-expressing transgenic mice.. Oncogene.

[44] Lueders KK, Kuff EL (1977). Sequences associated with intracisternal a particles are reiterated in the mouse genome.. Cell.

[45] Gray ER, Garson JA, Breuer J, Edwards S, Kellam P (2011). No Evidence of XMRV or Related Retroviruses in a London HIV-1-Positive Patient Cohort.. PLoS ONE.

[46] Kunstman KJ, Bhattacharya T, Flaherty J, Phair JP, Wolinsky SM (2010). Absence of xenotropic murine leukemia virus-related virus in blood cells of men at risk for and infected with HIV.. AIDS.

[47] Maggi F, Focosi D, Lanini L, Sbranti S, Mazzetti P (2011). Xenotropic murine leukaemia virus-related virus is not found in peripheral blood cells from treatment-naive human immunodeficiency virus-positive patients.. Clinical Microbiology and Infection.

[48] Barnes E, Flanagan P, Brown A, Robinson N, Brown H (2010). Failure to Detect Xenotropic Murine Leukemia Virus-Related Virus in Blood of Individuals at High Risk of Blood-Borne Viral Infections.. Journal of Infectious Diseases.

[49] Cornelissen M, Zorgdrager F, Blom P, Jurriaans S, Repping S (2010). Lack of Detection of XMRV in Seminal Plasma from HIV-1 Infected Men in The Netherlands.. PLoS ONE.

[50] Tang S, Zhao J, Viswanath R, Nyambi PN, Redd AD (2011). Absence of detectable xenotropic murine leukemia virus-related virus in plasma or peripheral blood mononuclear cells of human immunodeficiency virus Type 1-infected blood donors or individuals in Africa.. Transfusion.

[51] Lintas C, Guidi F, Manzi B, Mancini A, Curatolo P (2011). Lack of Infection with XMRV or Other MLV-Related Viruses in Blood, Post-Mortem Brains and Paternal Gametes of Autistic Individuals.. PLoS ONE.

[52] Satterfield B, Garcia R, Gurrieri F, Schwartz C (2010). PCR and serology find no association between xenotropic murine leukemia virus-related virus (XMRV) and autism.. Molecular Autism.

[53] Arnold RS, Makarova NV, Osunkoya AO, Suppiah S, Scott TA (2010). XMRV Infection in Patients With Prostate Cancer: Novel Serologic Assay and Correlation With PCR and FISH.. Urology.

[54] Fischer N, Schulz C, Stieler K, Hohn O, Lange C (2010). Xenotropic Murine Leukemia Virus-related Gammaretrovirus in Respiratory Tract.. Emerging Infectious Diseases.

[55] Sakuma R, Sakuma T, Ohmine S, Silverman RH, Ikeda Y (2010). Xenotropic murine leukemia virus-related virus is susceptible to AZT.. Virology.

[56] Singh IR, Gorzynski JE, Drobysheva D, Bassit L, Schinazi RF (2010). Raltegravir Is a Potent Inhibitor of XMRV, a Virus Implicated in Prostate Cancer and Chronic Fatigue Syndrome.. PLoS ONE.

[57] Nelson PN, Carnegie PR, Martin J, Davari Ejtehadi H, Hooley P (2003). Demystified. Human endogenous retroviruses.. Molecular Pathology.

[58] Balada E, Vilardell-Tarres M, Ordi-Ros J (2010). Implication of Human Endogenous Retroviruses in the Development of Autoimmune Diseases.. Int Rev Immunol.

[59] Lawoko A, Johansson B, Rabinayaran D, Pipkorn R, Blomberg J (2000). Increased immunoglobulin G, but not M, binding to endogenous retroviral antigens in HIV-1 infected persons.. J Med Virol.

[60] Moles JP, Hadi JC, Guilhou JJ (2003). High prevalence of an IgG response against murine leukemia virus (MLV) in patients with psoriasis.. Virus Research.

[61] Qiu X, Swanson P, Tang N, Leckie GW, Devare SG (2011). Seroprevalence of xenotropic murine leukemia virus-related virus in normal and retrovirus-infected blood donors.. Transfusion.

