# Supplementary figures and images for: Absence of XMRV in Peripheral Blood Mononuclear Cells of ARV-Treatment Naïve HIV-1 Infected and HIV-1/HCV Coinfected Individuals and Blood Donors
Source: PLoS One. 2012 Feb 13;7(2):e31398. doi: 10.1371/journal.pone.0031398 (PMC3278456; doi:10.1371/journal.pone.0031398)

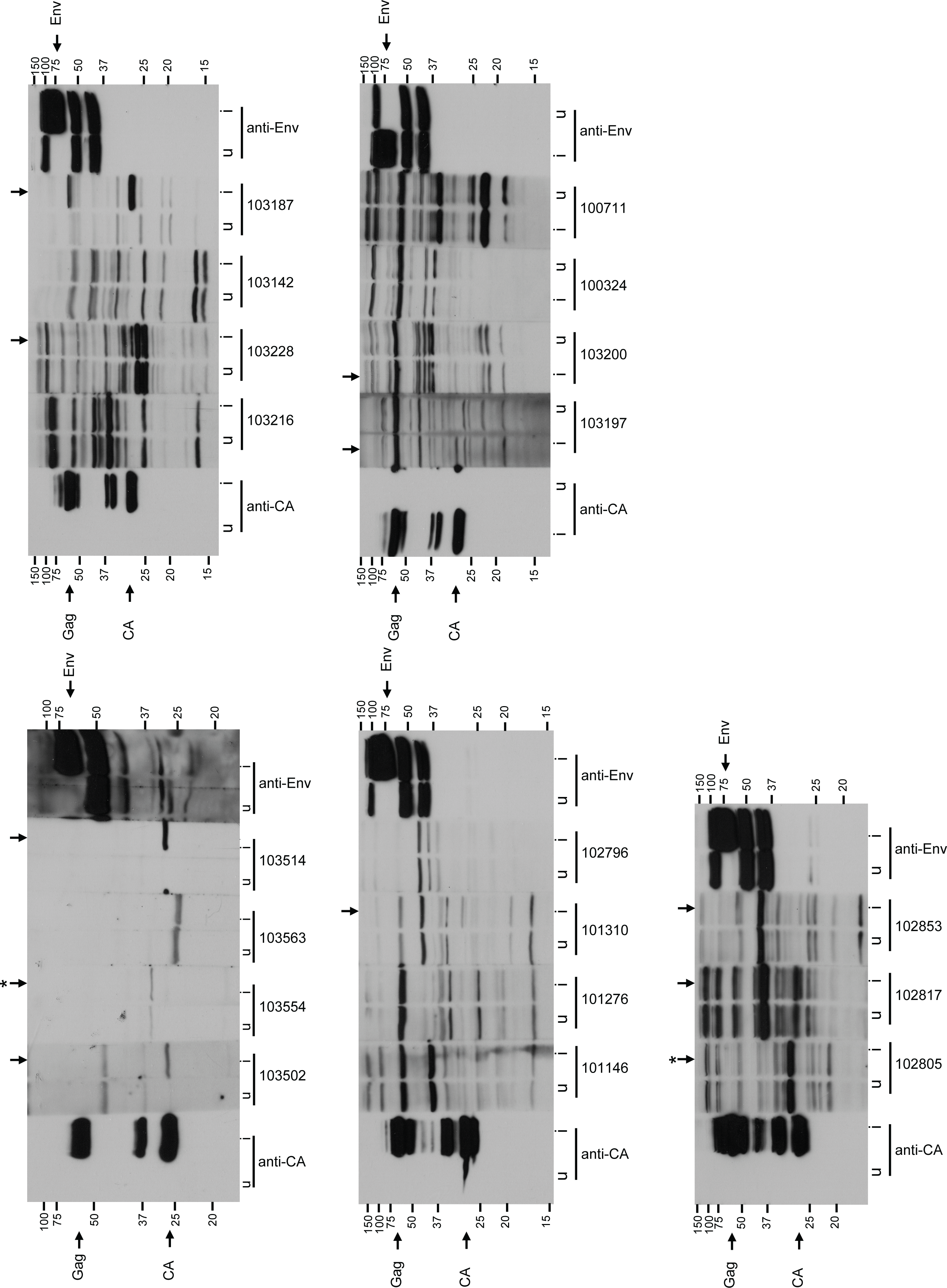

Supplement: Figure S1 — Screen for XMRV-reactive antibodies in HIV-1+ and HIV-1+/HCV+ patient sera. Western blots using uninfected (u) and XMRV-infected (i) LNCaP cell lysate as antigen for patient sera and positive-control antibodies against p30 capsid (anti-CA) and gp70 SU (anti-Env). Vertical arrows indicate lanes in which patient sera displayed reactivity to XMRV capsid. Protein mobilities are indicated in kiloDaltons. Vertical arrows with asterisks indicate lanes in which signals for XMRV-reactivity are more apparent with a longer film exposure. (TIF) [file pone.0031398.s001.tif]

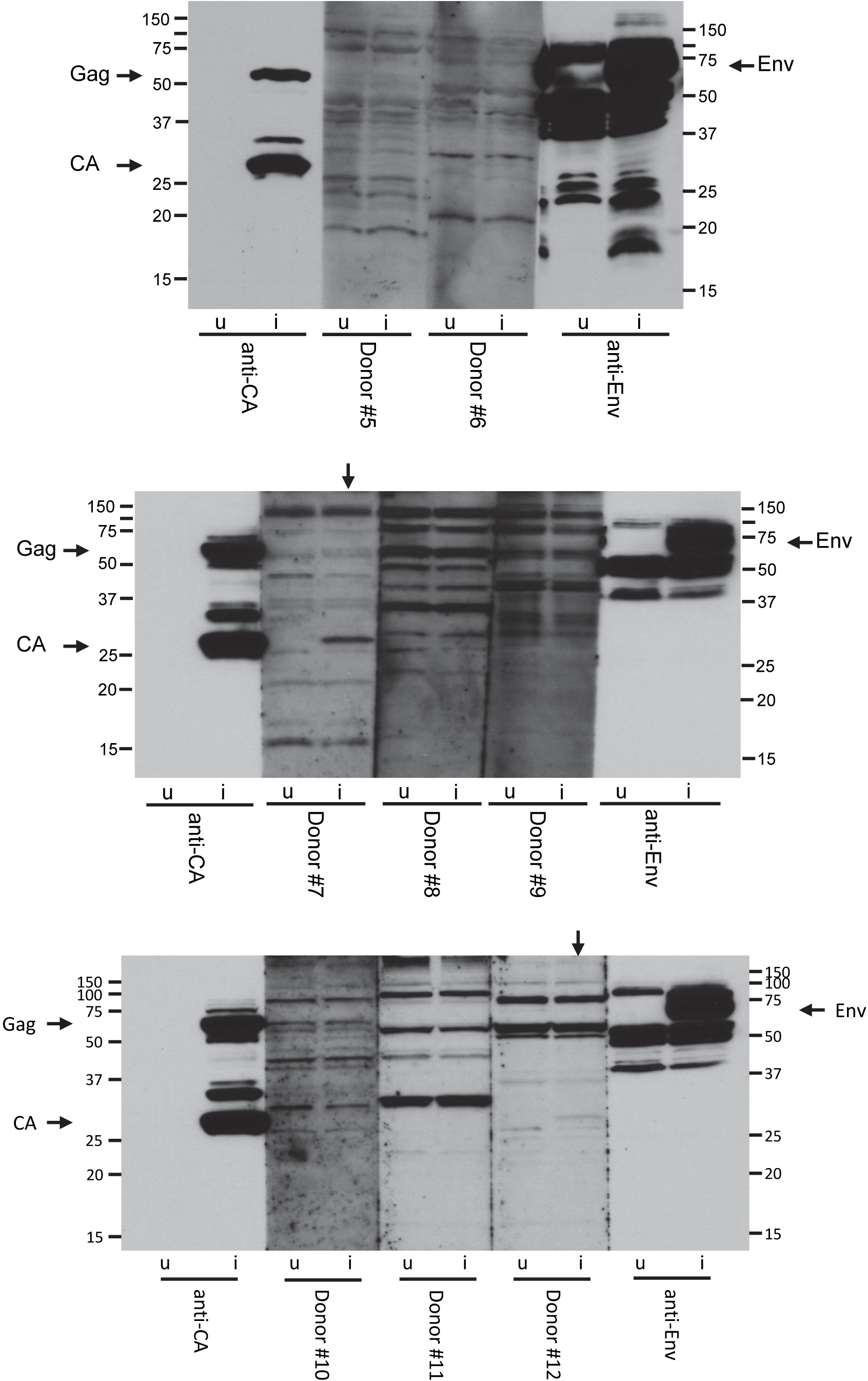

Supplement: Figure S2 — Screen for XMRV-reactive antibodies in healthy blood donors. Western blots using uninfected (u) and XMRV-infected (i) LNCaP cell lysate as antigen for healthy blood donor sera and positive-control antibodies against p30 capsid (anti-CA) and gp70 SU (anti-Env). Vertical arrows indicate lanes in which patient sera displayed reactivity to XMRV capsid. Protein mobilities are indicated in kiloDaltons. (TIF) [file pone.0031398.s002.tif]
